# Supplementary material for: Translational fidelity and longevity are genetically linked
Source: Nat Commun. 2025 Aug 13;16:7521. doi: 10.1038/s41467-025-62944-y (PMC12350961; doi:10.1038/s41467-025-62944-y)
Supplement: Supplementary file 1 — Supplementary Information [file 41467_2025_62944_MOESM1_ESM.pdf]

Translational Fidelity and Longevity are Genetically Linked

Boyang Zheng[1,2#], Weijie Zhang[1,2#], Gongwang Yu[3#], Wenjun Shi[2], Shuyun Deng[2], Xiaoyi Zhang[1,2], Jingyu Chen[2], Ziwei Zhou[1,2], Yuyan Shan[1,2], Wanting Wu[5], Erping Long[4], Xiaoshu Chen[1,5,6,7,*a], Jian-Rong Yang[1,2,6,7,*b]

[1] Advanced Medical Technology Center, The First Affiliated Hospital, Zhongshan School of Medicine, Sun Yat-sen University, Guangzhou 510080, China

[2] Department of Genetics and Biomedical Informatics, Zhongshan School of Medicine, Sun Yat-sen University, Guangzhou 510080, China

[3] The Affiliated Dongguan Songshan Lake Central Hospital, Guangdong Medical University, Dongguan 523326, China

[4] Institute of Basic Medical Sciences, Chinese Academy of Medical Sciences and Peking Union Medical College, Beijing 100005, China

[5] Department of Immunology and Microbiology, Zhongshan School of Medicine, Sun Yat-sen University, Guangzhou 510080, China

[6] Key Laboratory of Tropical Disease Control, Ministry of Education, Sun Yat-sen University, Guangzhou 510080, China

[7] Guangdong Provincial Highly Pathogenic Microorganism Science Data Center, Guangzhou 510440, China

[a] ORCID: 0000-0002-5779-5065

[b] ORCID: 0000-0002-7807-9455

[#] These authors contributed equally: Boyang Zheng, Weijie Zhang, Gongwang Yu

[*] Corresponding authors: Xiaoshu Chen (chenxshu3@mail.sysu.edu.cn), Jian-Rong Yang (yangjianrong@mail.sysu.edu.cn)

Supplementary Figures

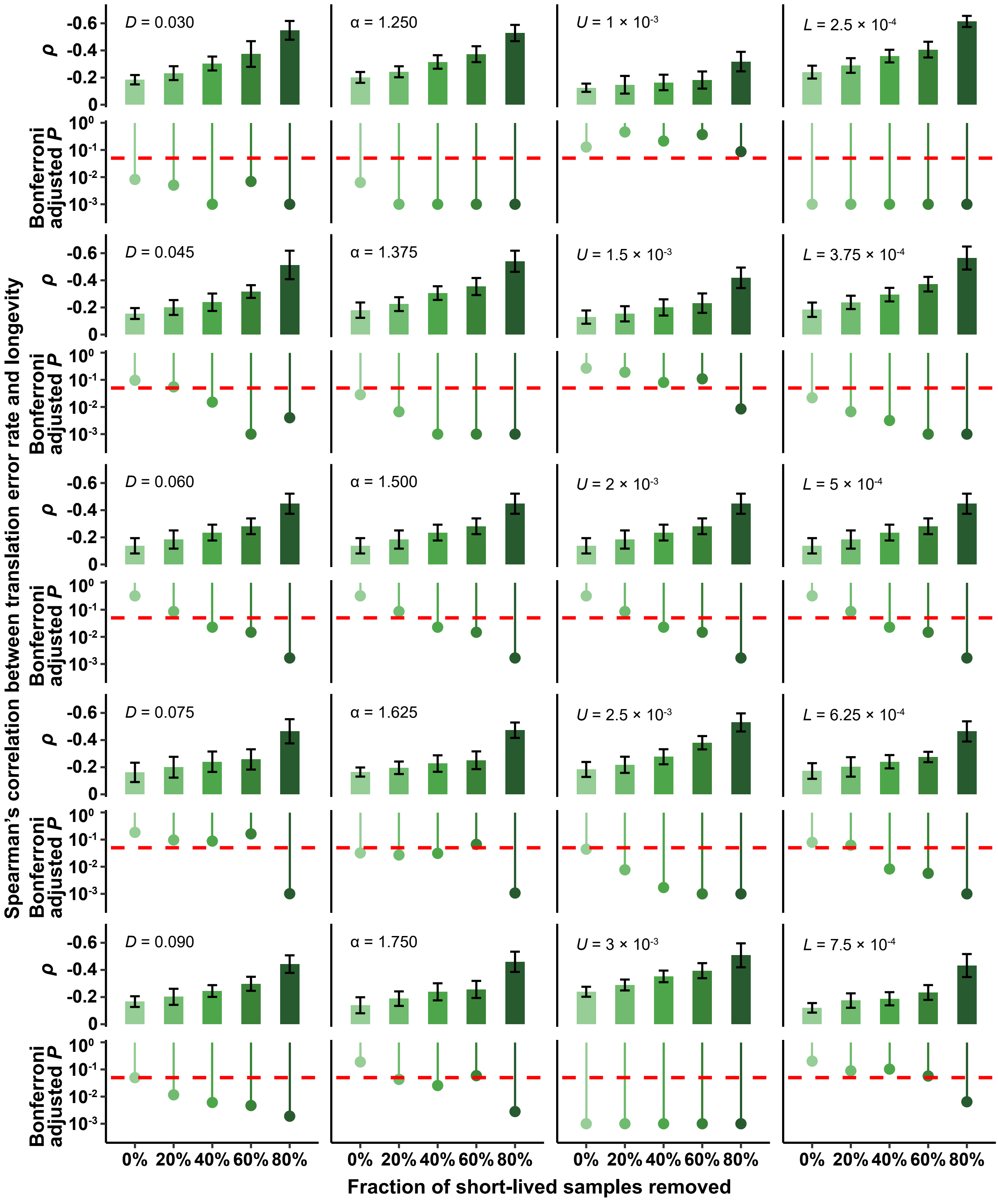

D = 0.030
α = 1.250
U = 1 × 10^-3
L = 2.5 × 10^-4
D = 0.045
α = 1.375
U = 1.5 × 10^-3
L = 3.75 × 10^-4
D = 0.060
α = 1.500
U = 2 × 10^-3
L = 5 × 10^-4
D = 0.075
α = 1.625
U = 2.5 × 10^-3
L = 6.25 × 10^-4
D = 0.090
α = 1.750
U = 3 × 10^-3
L = 7.5 × 10^-4
ρ
-0.6
-0.4
-0.2
0
Bonferroni adjusted P
10^0
10^-1
10^-2
10^-3
Spearman's correlation between translation error rate and longevity
0%
20%
40%
60%
80%
Fraction of short-lived samples removed

Supplementary Figure 1. The fidelity-longevity correlation among long-lived samples revealed by simulation is robust to changes in parameters.

Similar to Figure 1e except that each parameter (α, D, L, U) was individually changed. The changed parameters and value are indicated on the top left corner of each panel.

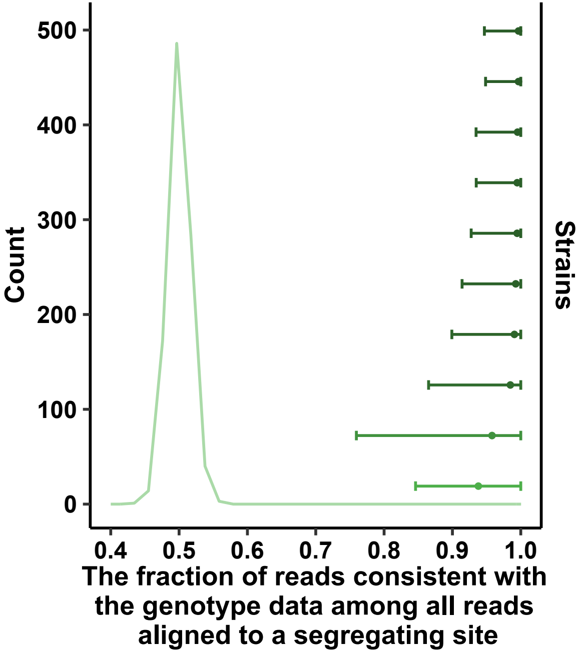

500
400
300
200
100
0
Count
Strains
0.4
0.5
0.6
0.7
0.8
0.9
1.0
The fraction of reads consistent with the genotype data among all reads aligned to a segregating site

Supplementary Figure 2. Resequencing result supports the quality of genotype data of the segregants.

Raw sequencing reads were first aligned to yeast genome, and then the fractions of reads consistent with the known genotype data (*x* axis) on every segregating sites were calculated and shown to the right. The mean fraction and its 95% confidence interval are respectively represented by the dot and the error bar. A control group of ten strains selected randomly from the segregants but not actually sequenced was similarly analyzed using the actual raw sequencing data. The curve to the left illustrates the overall distribution of the mean fraction (of reads consistent with the known genotype data of the control strains) from 1,000 such controls. The number of controls (*y* axis to the left) falling within each bin of width 0.02 was displayed. It appears that the actual genotype data have a much higher fraction than the random expectation, indicating their accuracy.

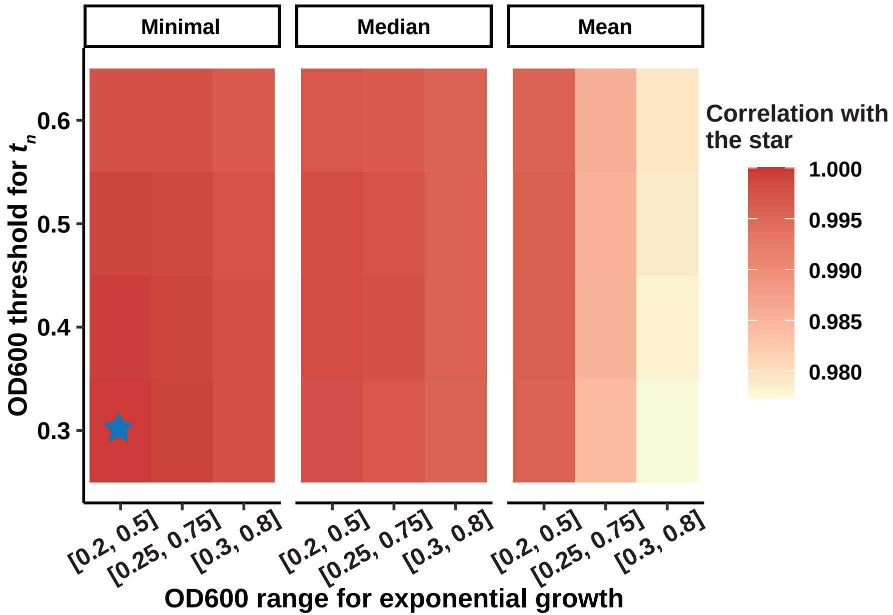
Minimal
Median
Mean
OD600 threshold for t_n
0.6
0.5
0.4
0.3
[0.2, 0.5]
[0.25, 0.75]
[0.3, 0.8]
OD600 range for exponential growth
Correlation with the star
1.000
0.995
0.990
0.985
0.980

Supplementary Figure 3. Accurate measurement of chronological lifespan.

Two-tailed Pearson’s correlation tests were conducted between the CLS presented in the main figures (blue star) and those estimated using other alternative settings (see Methods). These include (i) the range of OD600 considered as the exponential growth stage (x axis); (ii) the OD600 value used as the threshold for t_n (y axis); (iii) how the baseline doubling time (DT) is calculated using the DTs derived from the multiple ten-minute intervals during the exponential growth of subcultures on day 2 (the three panels). Each tile is colored based on Pearson's correlation, as indicated by the color scale bar at the right.

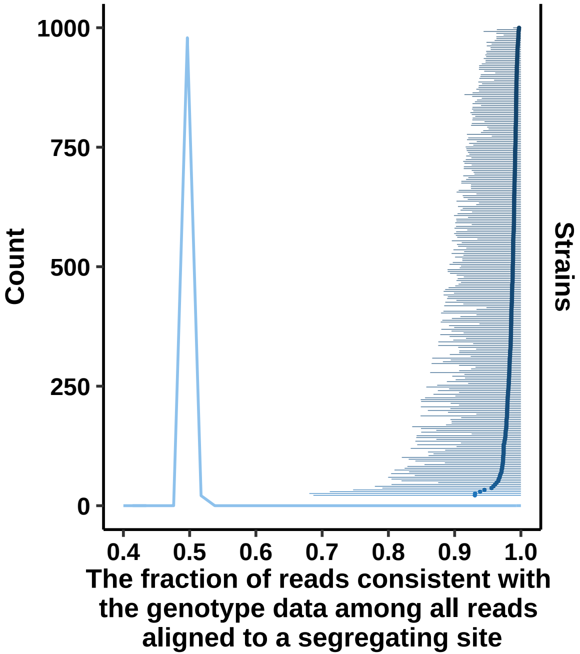
1000
750
500
250
0
Count
Strains
0.4
0.5
0.6
0.7
0.8
0.9
1.0
The fraction of reads consistent with the genotype data among all reads aligned to a segregating site

Supplementary Figure 4. Accuracy of genotype for the 260 strains carrying the dual luciferase system.

Similar to Supplementary Figure 2, except that the 260 strains carrying the dual luciferase system were resequenced. The control strains were created by randomly selecting 260 segregants.

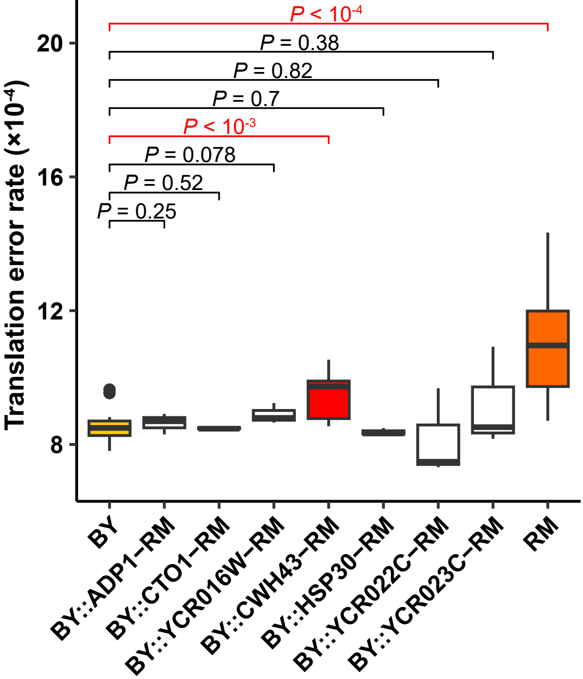

P < 10-4
P = 0.38
P = 0.82
P = 0.7
P < 10-3
P = 0.078
P = 0.52
P = 0.25
20
16
12
8
Translation error rate (×10-4)
BY
BY::ADP1−RM
BY::CTO1−RM
BY::YCR016W−RM
BY::CWH43−RM
BY::HSP30−RM
BY::YCR022C−RM
BY::YCR023C−RM
RM

Supplementary Figure 5. Identification of the translational fidelity-specific loci underlying the translation error rate of RM.

The parental RM strain exhibits higher translation error rate but also longer chronological lifespan, which is opposed to the prediction of the Error-Catastrophe Theory of Aging. This suggests the existence of other loci that regulate translation error rate (TER) and chronological lifespan independently (CLS). Based on the comparison between the QTL results of the two traits (i.e. Figure 5a), the TER-specific peak on chromosome III appears to be an important factor, since it only affects TER but not CLS. Therefore, we extracted all genes containing nonsynonymous SNPs between RM and BY in this peak, including *ADP1* (a putative ATP-dependent permease), *CTO1* (a protein required for cold tolerance), *YCR016W* (a RNA-binding ribosome assembly factor), *CWH43* (involved in GPI anchor biosynthesis and cell wall organization), *HSP30* (a heat shock protein), *YCR022C* (unknown function), and *YCR023C* (a vacuolar membrane protein of unknown function). We used a two-step method[57,58] to replace each of these genes in the BY parental strain with the same genes in the RM parental strain. It is found that when *CWH43* is replaced by its RM allele, the translation error rate is significantly increased by ~14.5% (median value across biological replicates, from 8.49×10^{-4} to 9.73×10^{-4}). This figure is similar to Figure 5f. The results are presented as standard box plots with the same criteria as in Figure 5e/f (n = 14, 3, 3, 3, 9, 3, 3, 3, 18 from left to right). *P* values from the one-tailed Wilcoxon rank-sum tests that compare the BY parental strain with each of the other strains are indicated on top. The two significant *P* values were highlighted red ($P = 4.4\times10^{-4}$ for BY::CWH43-RM and 4.1×10^{-7} for RM).

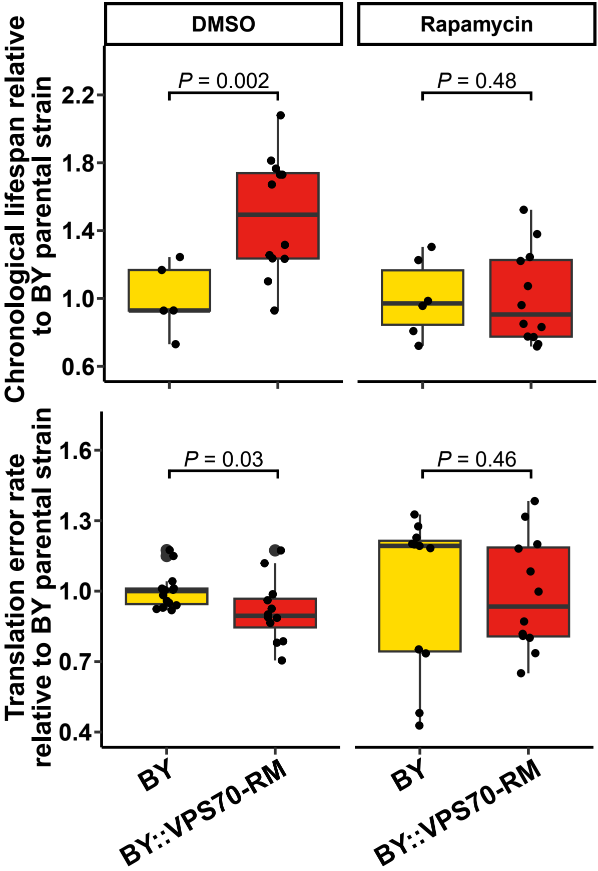
DMSO
Rapamycin
Chronological lifespan relative to BY parental strain
Translation error rate relative to BY parental strain
P = 0.002
P = 0.48
P = 0.03
P = 0.46
2.2
1.8
1.4
1.0
0.6
1.6
1.3
1.0
0.7
0.4
BY
BY::VPS70-RM
BY
BY::VPS70-RM

Supplementary Figure 6. Additional results on the function of *VPS70*.

Similar to Figure 5g, but with results from an independent set of experiments comparing DMSO and rapamycin treatments (for Rapamycin: n = 6 and 12 for the upper panel, and n = 11 and 12 for the lower panel; for DMSO: n = 5 and 12 for the upper panel, and n = 15 and 12 for the lower panel). The results are presented as standard box plots with the same criteria as in Figure 5g.

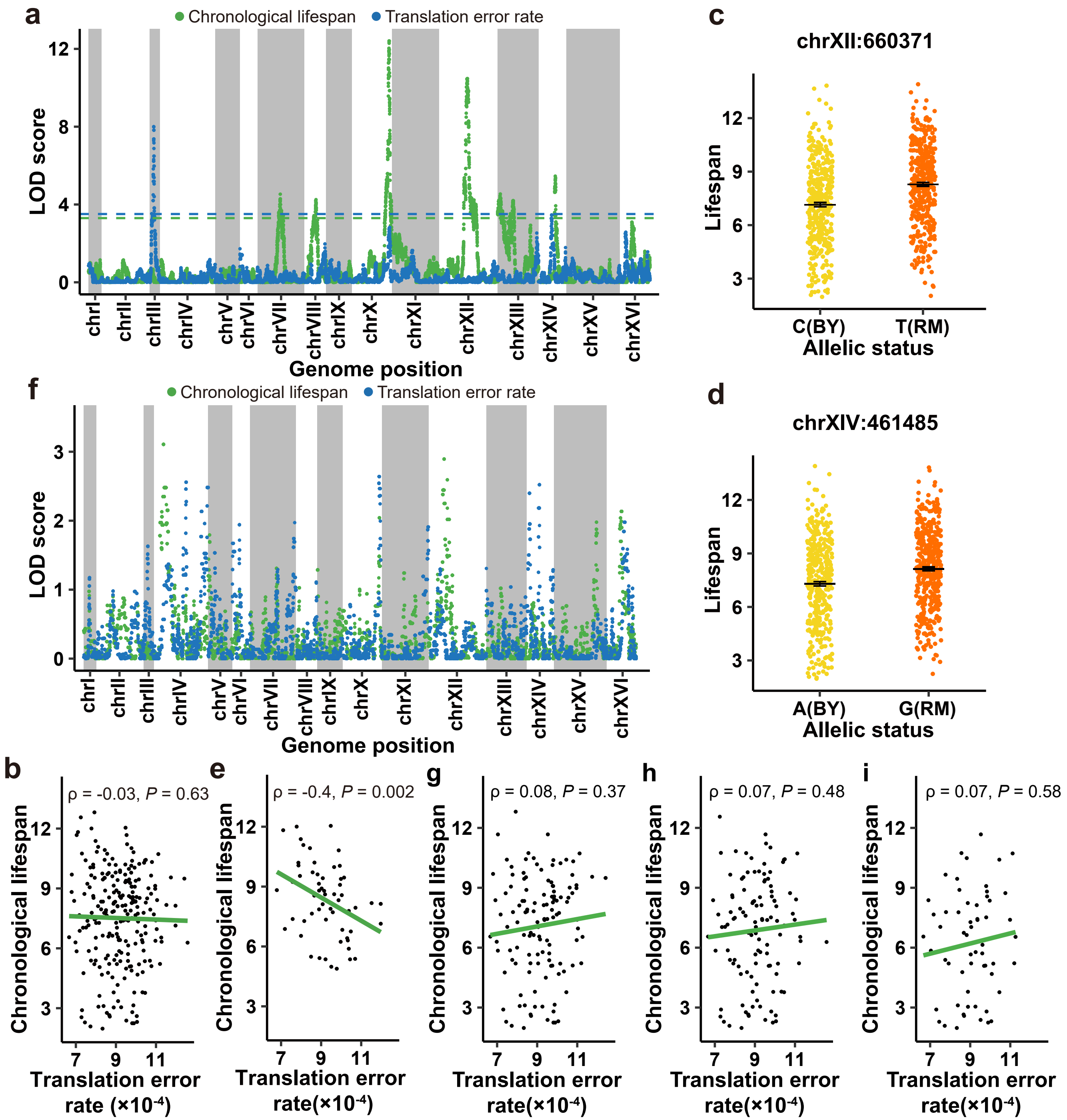
a
Chronological lifespan
Translation error rate
LOD score
Genome position
chrI chrII chrIII chrIV chrV chrVI chrVII chrVIII chrIX chrX chrXI chrXII chrXIII chrXIV chrXV chrXVI
c
chrXII:660371
Lifespan
C(BY)
T(RM)
Allelic status
f
Chronological lifespan
Translation error rate
LOD score
Genome position
d
chrXIV:461485
Lifespan
A(BY)
G(RM)
Allelic status
b
ρ = -0.03, P = 0.63
Chronological lifespan
Translation error rate (×10⁻⁴)
e
ρ = -0.4, P = 0.002
Chronological lifespan
Translation error rate(×10⁻⁴)
g
ρ = 0.08, P = 0.37
Chronological lifespan
Translation error rate(×10⁻⁴)
h
ρ = 0.07, P = 0.48
Chronological lifespan
Translation error rate(×10⁻⁴)
i
ρ = 0.07, P = 0.58
Chronological lifespan
Translation error rate(×10⁻⁴)

Supplementary Figure 7. The correlation between lifespan and translation error rate could also be demonstrated by controlling the second and third most significant QTLs for lifespan.

a Manhattan plot of QTL mapping results. The green symbol represents the result for chronological lifespan using all 804 strains with data available. The blue symbol represents the result for translation error rate using all 260 strains with available data.

b No correlation between chronological lifespan and translation error rate was found when all strains with available data were analyzed (n = 235). Spearman's Correlation Coefficient and corresponding *P* value are indicated (two-tailed Spearman's correlation test).

c and **d** Association between the allelic state (*x* axis) and the chronological lifespan (*y* axis) is shown for the one SNP with the highest LOD score in the second (**c**) or third (**d**) most significant peak in **a** (n = 804). Error bars represent standard errors of the mean.

e Similar to **b**, but controlled the second and third most significant QTLs by focusing on strains with Thymine on chrXII:660,371 and Guanine on chrXIV:461,485 (n = 58). Spearman's Correlation Coefficient and corresponding *P* value are indicated (two-tailed Spearman's correlation test).

f Same as Figure 5a, except for controlling the second and third most significant QTLs. An overlapping peak between chronological lifespan and translation error rate could be seen on chrX.

g and **h** No correlation between chronological lifespan and translation error rate was found when segregants having the BY alleles on ChrXII:660,371 and on ChrXIV:461,485 were analyzed, respectively. Spearman's Correlation Coefficient and corresponding P value are indicated.

i Same as **e**, but for segregants with BY alleles on both ChrXII:660,371 and ChrXIV:461,485.
