# Supplementary material for: Translational fidelity and longevity are genetically linked
Source: Nat Commun. 2025 Aug 13;16:7521. doi: 10.1038/s41467-025-62944-y (PMC12350961; doi:10.1038/s41467-025-62944-y)
Supplement: Supplementary file 2 — Description of Additional Supplementary Files [file 41467_2025_62944_MOESM2_ESM.pdf]

Description of Additional Supplementary Files

Supplementary Data 1. List of strains included/excluded by quality filters.

Supplementary Data 2. Chronological lifespan and translation error rate of 235 segregant strains.

Supplementary Data 3. Primer sequences used in this study.
